# Optimizing Sensor Locations for Electrodermal Activity Monitoring Using a Wearable Belt System ^†^

**DOI:** 10.3390/jsan14020031

**Published:** 2025-03-18

**Authors:** Riley Q. McNaboe, Youngsun Kong, Wendy A. Henderson, Xiaomei Cong, Aolan Li, Min-Hee Seo, Ming-Hui Chen, Bin Feng, Hugo F. Posada-Quintero

**Affiliations:** 1 Department of Biomedical Engineering, College of Engineering, University of Connecticut, Storrs, CT 06269, USA; 2 Department of Biobehavioral Health Sciences, School of Nursing, University of Pennsylvania, Philadelphia, PA 19104, USA; 3 School of Nursing, Yale University, Orange, CT 06477, USA; 4 Department of Statistics, College of Liberal Arts and Sciences, University of Connecticut, Storrs, CT 06269, USA; 5 Institute of Materials Science, University of Connecticut, Storrs, CT 06269, USA

**Keywords:** electrodermal activity, wearable sensor, torso, optimal location

## Abstract

Wearable devices for continuous health monitoring in humans are constantly evolving, yet the signal quality may be improved by optimizing electrode placement. While the commonly used locations to measure electrodermal activity (EDA) are at the fingers or the wrist, alternative locations, such as the torso, need to be considered when applying an integrated multimodal approach of concurrently recording multiple bio-signals, such as the monitoring of visceral pain symptoms like those related to irritable bowel syndrome (IBS). This study aims to quantitatively determine the EDA signal quality at four torso locations (mid-chest, upper abdomen, lower back, and mid-back) in comparison to EDA signals recorded from the fingers. Concurrent EDA signals from five body locations were collected from twenty healthy participants as they completed a Stroop Task and a Cold Pressor task that elicited salient autonomic responses. Mean skin conductance (meanSCL), non-specific skin conductance responses (NS.SCRs), and sympathetic response (TVSymp) were derived from the torso EDA signals and compared with signals from the fingers. Notably, TVSymp recorded from the mid-chest location showed significant changes between baseline and Stroop phase, consistent with the TVSymp recorded from the fingers. A high correlation (0.77–0.83) was also identified between TVSymp recorded from the fingers and three torso locations: mid-chest, upper abdomen, and lower back locations. While the fingertips remain the optimal site for EDA measurement, the mid-chest exhibited the strongest potential as an alternative recording site, with the upper abdomen and lower back also demonstrating promising results. These findings suggest that torso-based EDA measurements have the potential to provide reliable measurement of sympathetic neural activities and may be incorporated into a wearable belt system for multimodal monitoring.

## 1. Introduction

Over the past two decades, the use of wearable devices has increased significantly to meet the demand for reliable, continuous health monitoring. The integration of multiple sensors into a single device has facilitated the simultaneous acquisition of multiple bio-signal measurements from a single, unobtrusive, and localized site, such as the wrist. Electrodermal activity (EDA) that quantifies skin conductance is a critical modality of measurement of these devices as it provides invaluable insight into sympathetic nervous system (SNS) activity and its correlation with emotional arousal [1], fatigue [2], pain [3–5], and other physiological parameters. In general, an EDA signal recorded from the fingers or wrist provides the best balance of signal fidelity and practicality [6–8]. Recording EDA from other parts of the body requires systematic characterization and optimization due to its intrinsic positional sensitivity. EDA may be a potential indicator of visceral pain associated with irritable bowel syndrome (IBS) and other health conditions, correlating with the dysregulated autonomic nervous system. The characteristic symptoms of IBS include visceral hypersensitivity and altered pain perception, which manifest as abdominal cramping, discomfort, or pain [9]. A system for effective monitoring of pain in IBS patients requires the recording of multiple bio-signals sensitive to IBS-related symptoms, such as EDA, electrocardiogram (ECG), and electromyogram (EMG). For practical implementation, this proposed system would ideally acquire all signals from a single location to reduce user burden, while considering the scope and limitations of each modality. The most limiting signal is EMG due to the location-specific requirements of the signal in monitoring abdominal contractions. ECG exhibits spatial flexibility for electrode placement, enabling reliable cardiac peak detection from multiple sites, including the torso. EDA response is primarily moderated by body-wide innervation of sweat glands via the sympathetic nervous system, theoretically providing flexibility in its acquisition site. However, comprehensive validation of the torso as an optimal site for EDA measurement remains to be established.

Exploring alternate locations on the body for EDA signal acquisition is not a novel undertaking. There is a general consensus that the finger pads and/or palmar surfaces are the optimal location for EDA acquisition due to the high density of eccrine sweat glands that yield high-quality signals [6,10,11]. It has been proposed that the density of glands in these areas can be readily linked to emotions due to their relation to grasping and escaping abilities [12]. Eccrine glands in other locations, which serve primarily thermoregulatory roles, may share similar EDA profiles. In turn, many groups have sought out these alternatives for various reasons. For example, the shoulder, forearm, and lower calf have all been considered as substitutes to facilitate practical, long-term EDA monitoring [13,14]. Various locations on the head have been evaluated as well, such as the forehead and the neck to circumvent motion artifacts related to finer movement and propose alternative use cases for the signal [13–15]. A previous study by van Dooren et al. [13] investigated regions of the torso for EDA recordings, including the back, abdomen, and chest. Results indicated a low correlation between the torso EDA signals and signals from the fingers while subjects were presented with visual stimuli of emotional videos. However, the study compared EDA signals in the form of skin conductance level (SCL)/skin conductance responses (SCRs) and could benefit from additional advanced data processing. For example, a time–frequency index of sympathetic activity for EDA, TVSymp, shows greater sensitivity to stimuli in comparison to traditional, time-domain metrics like SCL [16]. Rigorous processing of EDA from the torso using such metrics will potentially enhance the informative nature of the torso-derived signal.

In this study, we conducted concurrent EDA recordings from both the finger and torso regions of healthy volunteers undergoing a Stroop task and a Cold Pressor task. We completed thorough signal processing to derive representative metrics of mean skin conductance (meanSCL), non-specific skin conductance responses (NS.SCRs), and sympathetic response (TVSymp). We then compared indices between EDA measured from different regions of the torso and the fingers. This paper highlights the experimental approach and its motivation, presents the derivation and summary of signal metrics and concludes with a rigorous discussion comparing stimulus sensitivity and metric correlation for the exploratory locations along with commentary on protocol limitations.

## 2. Materials and Methods

**Figure 1.**
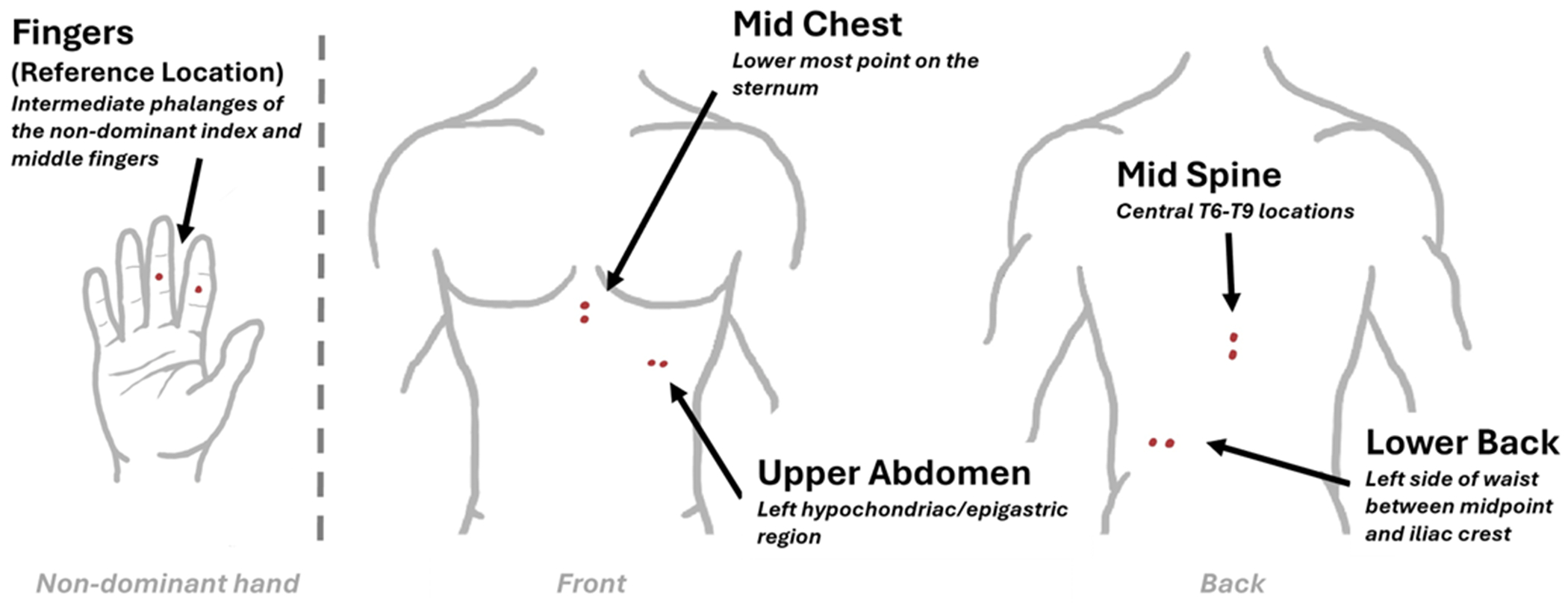
A map of the four exploratory torso-based sites selected for EDA acquisition. The fingers were used as a reference. Red dots indicate electrode pairs and their orientation.

The present study investigates the quality of EDA acquired from different locations on the torso. Four different locations were considered: the mid-chest, upper abdomen, lower back, and mid-back (see Figure 1). These locations were selected from possible torso-based options according to their sweat rate, estimated sweat gland density, and similarity to previous studies, allowing for a meaningful comparison [11,13,17]. Visceral pain manifests through SNS arousal, which can be quantified via electrodermal activity analysis [5,16]. To effectively explore potential sites for capturing SNS activation on the torso, we selected two established stimuli known to modulate autonomic nervous system responses, while acknowledging the limitations in directly replicating IBS-specific visceral pain paradigms. A Stroop task was employed to elicit an emotional response that was reported to correlate with changes in EDA activity in our previous study [18]. A Cold Pressor task was used to capture the response to painful stimuli, which also produces changes in EDA levels relative to the applied stimulus intensity [3]. The EDA recorded from fingers was used as a reference both for comparing the detection sensitivities and conducting correlation analyses with torso-based EDA recordings. A previously published conference paper presented initial results [19]. This paper expands upon the original approach by incorporating more subjects in the analysis demonstrating novel results not initially present in signal detection sensitivity. It similarly incorporates an improved comparison of location correlation along with a detailed review of previous literature and an involved discussion of results/limitations.

### 2.1. Participants

Twenty healthy subjects (11 males, 9 females) ranging in age from 18 to 32 were enrolled in the study after providing formal written consent. The protocol and consenting processes were approved by the Institutional Review Board for human subject research at the University of Connecticut. Prior to their laboratory visit, all participants were instructed to abstain from caffeine and other stimulant substances for a period of 24 h. Five subjects were excluded from the study due to the recorded skin conductance exceeding the cut-off limits of 0.2–100 μS, indicative of poor signal quality likely from disconnected electrodes on the torso. The cutoff was defined by the parameters of the acquisition system specifications. The five corrupted subjects had frequent and consistent saturation of the lower back, which most often resulted from electrodes that became detached over the course of the protocol. The total number of subjects included in the study was 15.

### 2.2. EDA Acquisition

EDA was simultaneously acquired using a set of commercially available wearable devices (Shimmer3+ GSR Unit, Shimmer, Dublin Ireland). Signals were sampled at 120 Hz while a proprietary laboratory-developed application was used to collect, synchronize, and monitor the recordings. Self-adhesive, ConMed gel-based Ag/AgCl electrodes (CONMED Corp. Largo, FL, USA) were placed on five different locations across the body including the fingers (index and middle fingers of non-dominant hand), mid-chest, upper abdomen, lower back, and mid-back as illustrated in Figure 1. Each area was cleaned with 70% isopropyl alcohol (Vi-Jon Laboratories, St. Louis, MO, USA) prior to placing the electrodes and shaved if necessary. The orientation and distance of the electrodes were kept fixed for all participants. The Shimmer devices were secured to the body near the respective electrodes using medical tape. Any loose wires were secured with tape as well to reduce the noise induced by abrasion with clothing/movement.

### 2.3. Protocol

The experiment was conducted in a dimly lit, private laboratory setting with only the experimenter and participant present. A female research assistant aided in applying electrodes for female subjects. The staff member cleaned and placed the electrodes on the four torso locations and the fingers. Recordings of EDA started immediately after the electrodes were connected to the wearable sensors, and the signal quality was validated.

**Figure 2.**
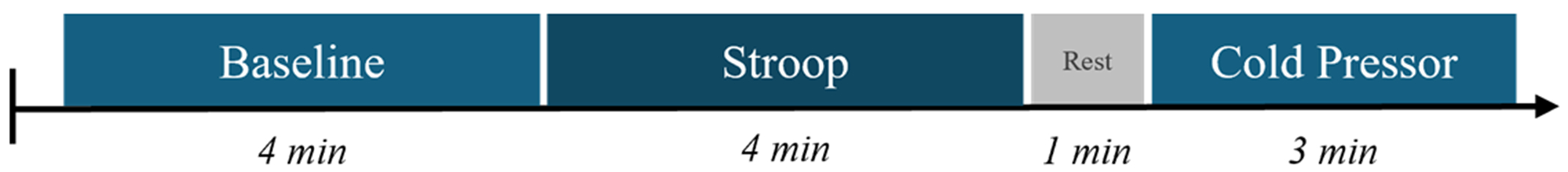
Timeline of the protocol used in data collection.

The experiment was divided into three phases, which were completed as the subject sat upright in a stationary chair. The subject was required to ensure that their back was not in contact with any part of the chair while sitting as still as possible to prevent undesirable motion artifacts. The initial Baseline phase comprised a four-minute period during which the participants were instructed to silently relax with their eyes open. The second phase, the Stroop task, consisted of a four-minute Stroop task that the participants completed on a tablet suspended in front of them immediately following the baseline. Four minutes was determined as a reasonable time window to elicit an EDA response as responses have been seen in as little as 40–120 s [18,20]. They were instructed to vocally indicate the color in which the text on the screen was written. The third phase, the Cold Pressor task, required the participants to submerge their dominant hand in an ice-cold reservoir maintained at a temperature of 4 °C for a minimum duration of three minutes. A 60 s break separated the Stroop from the Cold Pressor to let the subject rest and allow time to position the necessary ice water bath. Following the completion of all three phases, the recordings were concluded. A summary of the protocol is displayed in Figure 2.

### 2.4. Data Processing

The raw EDA data were resampled at a rate of 8 Hz and subsequently filtered using a 1 Hz cutoff low-pass filter, a standard processing procedure that isolates the most informative frequencies related to EDA and reduces computational load [21]. The filtered signal was then decomposed into tonic and phasic series using the cvxEDA algorithm [22]. TVSymp, the time-variant feature described previously, was also acquired. The metric is calculated by further down sampling the EDA signal to 2 Hz and obtaining the spectral components of the signal between 0.01 and 0.26 Hz using variable complex demodulation (VFDCM) to isolate the frequencies most representative of sympathetic activity. The resulting time-varying spectral amplitudes *X*′(*t*) were then summed, normalized, and compiled using a Hilbert transform to acquire its instantaneous amplitude as seen in Equations (1)–(3) as follows,

(1) $$Y(t)=\frac{1}{\pi }P\int \frac{X(\tau )}{t-\tau }d$$

(2) $$Z(t)=X(t)+iY(t)=a(t)e^{i\phi (t)}$$

(3) $$a(t)={\left[X^{2}(t)+Y^{2}(t)\right]}^{1/2},\theta (t)=\arctan\left(\frac{Y^{\prime }(t)}{X^{\prime }(t)}\right)$$

where *P* indicates the Cauchy principal value, X′(t) and Y′(t) are the complex conjugate pairs, *Z*(*t*) is the analytic signal, and *a*(*t*) is the final instantaneous amplitude of which TVSymp is the mean amplitude. Note that a detailed summary of the metric, the VFCDM algorithm, and its supporting calculations can be found in a previous paper [16].

The resulting three waveforms were then segmented to match the three separate phases of the protocol using digitally annotated event markers. For each phase, three distinct metrics were derived, two of which were the mean skin conductance represented by the mean of the tonic signal (meanSCL) and the number of non-specific skin conductance responses from the phasic (NS.SCRs) using a 0.05 μS threshold. Such skin conductance responses are those rapid and smooth transient events in the EDA signal that do not specifically correspond to any single stimulus. The remaining metric was the mean value of the TVSymp waveform.

### 2.5. Statistical Analysis

Two primary relationships were explored in the statistical analysis. First, the derived metrics were compared across the three phases in Figure 2 using a paired *t*-test with Bonferroni correction for multiple comparisons to compare measurement sensitivity to applied stimuli. Statistical significance was set a priori at *α* = 0.05. Second, the Pearson correlation was between sample means of measurements computed from four torso locations and measurements from the fingers. This approach was selected as many studies have found that the correlation of raw waveforms has the potential to over or under estimate the similarity of different signals for EDA [15,23].

## 3. Results

**Figure 3.**
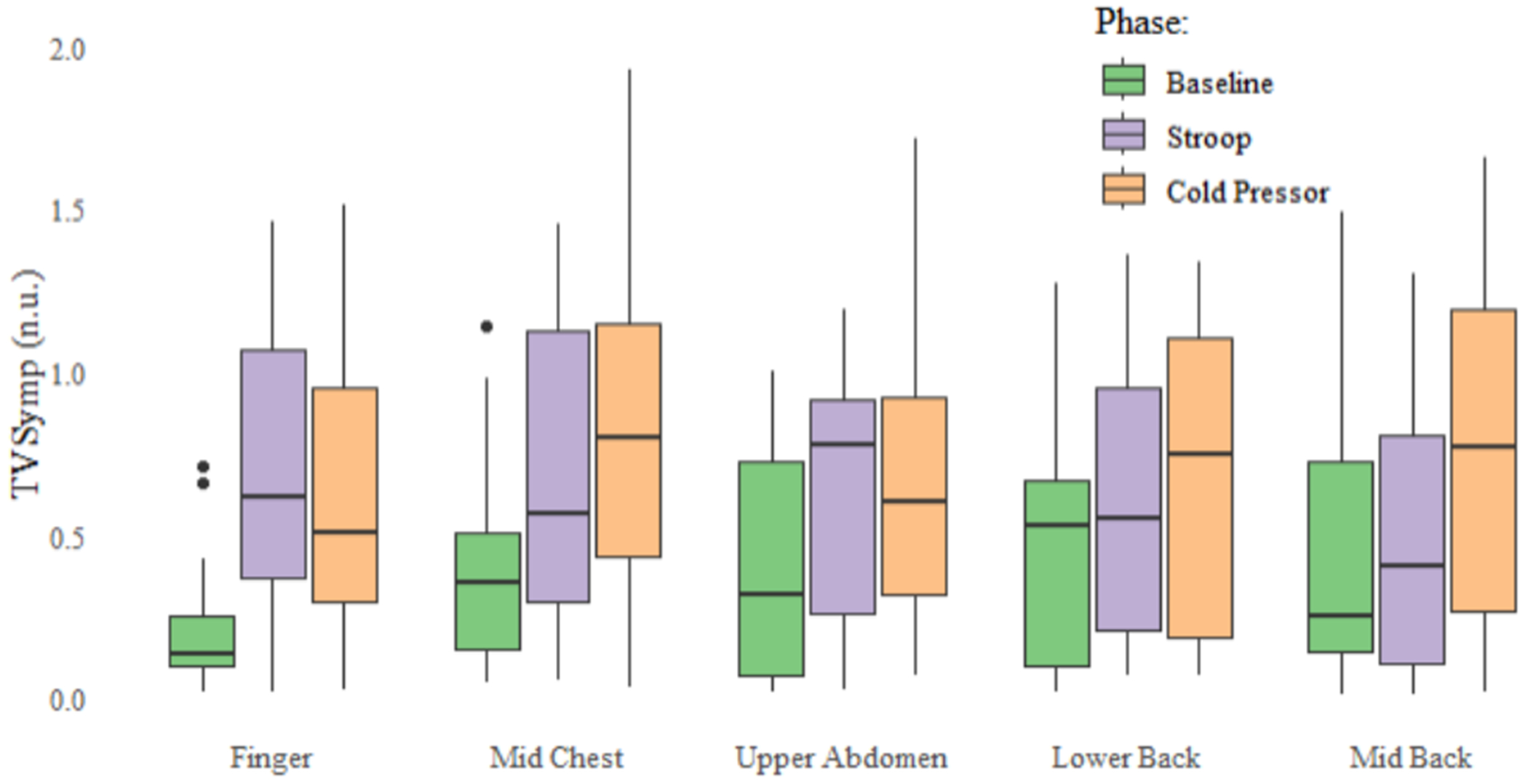
Comparison of TVSymp across the five locations for all three phases of the protocol. Black dots indicate outliers.

Table 1 contains the metrics derived for the study participants across all protocol phases and locations. The EDA signals from the fingers, i.e., reference location, exhibited statistically significant differences in the NS.SCR count between the baseline (M = 57.08, SD = 21.28) and Stroop phases (M = 75.098, SD = 16.82) (*p* < 0.05). This statistical significance is not replicated in any of the test locations on the torso for NS.SCRs. The TVSymp recorded from the fingers also showed significant differences in mean TVSymp values between the baseline (M = 0.255, SD = 0.24) and both the Stroop (M = 0.813, SD = 0.45, *p* < 0.02) and Cold Pressor (M = 0.737, SD = 0.43, *p* < 0.02) phases. TVSymp derived from the mid-chest location also demonstrates sensitive detection of the three test phases in Figure 2, showing significant difference in average TVSymp between baseline (M = 0.394, SD = 0.37) and Cold Pressor (M = 0.878, SD = 0.61) (*p* < 0.01) phases. Similar trends amongst the metrics, albeit not significant, are visualized in Figure 3 across other torso locations. Within mean SCL, the fingers, upper abdomen, and lower back all showed sequential increases across the protocol while the mid-chest and mid-back decreased for the Stroop task. Regarding NS.SCRs, signals recorded from the torso locations exhibited different trends as compared to the fingers, which showed a decreased activity after an initial increase during the Stroop task. On average, TVSymp from the fingers increased from the baseline phase to the Stroop task but decreased during the Cold Pressor while the rest of the locations showed sequential increases across the protocol timeline, again visible in Figure 3.

Table 2 presents the correlation coefficients for the comparison of the aggregate waveforms for the test locations to the reference finger site across all phases of the protocol. Comparing the sample means of the meanSCL across the three phases demonstrated a high correlation to recordings from the fingers for the upper abdomen and lower back locations at 0.96 and 0.78, respectively. The correlation of mean NS.SCRs across the protocol was highest for the lower back at 0.91. Correlations based on sample means for TVSymp across the three phases consistently showed high correlation for the mid-chest, upper abdomen and lower back at 0.77, 0.78, and 0.83, respectively.

## 4. Discussion

The objective of this study was to assess the quality of electrodermal activity (EDA) signals recorded from four distinct locations of the torso, which is driven by the needs of integrating EDA recordings into a wearable belt system for multimodal bio-signal recordings. The belt is strategically located around the lower torso for detecting visceral pain associated with IBS. The selection of these torso locations was informed by several factors, including eccrine sweat gland density, sweat maps, prior torso-based EDA tests, and practical considerations. The mid-chest, upper abdomen, back hip, and mid-back were identified as potential locations for reliable recording of EDA, with the fingers serving as the gold standard for reference [6]. To evaluate EDA signal quality from those locations, participants completed two behavioral tasks, i.e., the Stroop and Cold Pressor tasks that induced cognitive and physical stresses, respectively.

The EDA recorded from the fingers, the reference location, demonstrated expected detection sensitivity to the stimulus tasks, showing a significant difference in NS.SCRs and TVSymp before and after the tasks. Importantly, EDA recorded from mid-chest location also possesses sufficient detection sensitivity showing a significant increase in mean TVSymp values during the Cold Pressor task in comparison to the baseline. In addition, there was significant positive correlation between the EDA signals recorded from mid-chest and from fingers, indicating similar signal trends across varying stimuli. The absence of statistical significance when analyzing meanSCL and NS.SCRs for the mid-chest location may underscore the necessity for advanced signal processing with EDA signals. The aforementioned findings demonstrate the highly sensitive nature of TVSymp in detecting differences in applied stimuli. Consequently, the metric emerges as a valuable instrument in the exploration of alternate locations indicative of specific EDA traits, superseding the reliance on correlations of basic EDA waveforms.

MeanSCL and NS.SCR measures from the mid-back had relatively low correlation to the fingers at −0.10 and 0.02, respectively. A similar pattern is reflected, albeit less intensely, by the mid-chest at 0.58 and 0.58, respectively. EDA from these locations may be more susceptible to noise such as respiration artifacts or minor motion artifacts resulting from their intrinsic positioning higher up on the torso. They are also both found in moisture retentive regions, potentially owing to changes in measured skin conductance to sweat pooling rather than SNS sweat pore modulation, which EDA seeks to capture. These considerations may not be as relevant for EDA from the upper abdomen and lower back, sites with higher correlations that are located closer to the waist and away from sweat accumulating regions.

While the correlations are relatively high, the varying trends in the EDA response between the finger and torso locations for all metrics across the protocol phases, specifically their inability to reflect a decrease in NS.SCR and TVSymp during the Cold Pressor task and after the Stroop task, may indicate insensitivity to specific stimuli. While the mid-chest and other torso locations exhibited general activation across the stimulus phases, it is imperative that any location selected for EDA signal acquisition be capable of discerning both activation and relaxation periods of the sympathetic system to ensure the acquisition of meaningful information.

It is crucial to note the high standard deviations and broad confidence intervals across many of the torso locations, especially during the Stroop task for meanSCL and TVSymp. This variability is not present in the metrics derived from the fingers and may indicate high inter-subject variability when recording from the torso. While the presence of a stimulus may be detectable, its relative EDA effect size could be reduced due to location specific differences such as skin composition and other anatomical factors.

The observed differences in response between the fingers and the torso locations can be attributed to a range of different factors. Notably, the densities of sweat glands on the torso and variations in skin composition can modulate the presentation of the EDA waveform. Skin hydration and surface level moisture readings can vary across the body and are difficult to control across participants, resulting in high variability in long-term EDA recordings. Additionally, involuntary torso movements that are absent in the fingers such as stomach contractions or respiration may also further confound the EDA signals.

The results reported herein show significant insights into the quality of electrodermal activity (EDA) recorded from the torso. The detection sensitivity of EDA from the torso locations appears to be inferior to that from the fingers, also the EDA from the torso was unable to detect the reduction period of the sympathetic tone. Nonetheless, the reliable detection of the activation of the sympathetic tone by torso EDA signals can be valuable when combined with signals from other sources, such as EMG and ECG. It is noteworthy that the mid-chest region demonstrated a degree of sensitivity to stimuli that was commensurate with that observed in the fingers during the analysis of TVSymp responses. Shared differences trending toward significance with notable correlations from the lower back and upper abdomen may also indicate potential alternates given further investigation and study improvements. When selecting an optimal location, it is imperative to consider the practicality of these three options. A salient concern for the mid-chest or abdomen locations is the need to consider the effects of body shape on their practical implementation in the belt. In light of these considerations, the lower back emerges as a potentially optimal alternative. The ability to collect EDA from the torso enables a broad spectrum of applications that extend beyond the realm of IBS. These applications include the integration of EDA monitoring into chest-based devices utilized for the surveillance of health concerns such as chronic lung diseases [24] or high-risk cardiac patients [25].

The present study is not without its limitations. One possibility is that the limited response observed at many locations may be due to protocol-specific factors, such as the duration, intensity, and/or difficulty of Stroop and Cold Pressor tasks. While the stimulus duration utilized followed normal procedures seen in many previous works, high variability in the results may indicate the need for more rigorous stimulus paradigms. The torso locations may not be as sensitive in capturing SNS responses to mild stimuli. Moving forward, it is essential to test IBS-specific stimuli when evaluating these locations to ensure the duration and intensity are sufficient to elicit a significant EDA response in the intended scenario. As previously mentioned, the substantial variability in outcomes could be attributed to differences in skin hydration levels. The ability of certain sites to exhibit electrodermal activity depends on adequate hydration, which is influenced by both skin location and environmental conditions. Insufficient hydration may introduce variability amongst subject responses between baseline and arousal readings [23]. In the present study, skin moisture was not assessed as part of the experimental design, a limitation frequently found in many EDA studies. In future research, incorporating methods that seek to control or monitor skin hydration/perspiration may provide relevant data to improve results. While not extensively explored, many studies have noted the effect of gender and race on EDA recordings, factors which may provide further clarity on differing results [26,27]. Finally, we did not account for the type of clothing worn over the devices, which may have had varying, negative effects by interacting with the secured electrodes and sensors. This most likely contributed to the corrupted subject data excluded from the analysis. Without controlling clothing, the employment of better techniques to secure the electrodes to the torso may improve data collection and reduce the presence of unusable, corrupted data. As five subjects had to be excluded due to poor signal quality, increasing protocol resilience should be of the utmost importance. Optimizing signal fidelity may be achieved through strategic refinements in device and electrode securement and design. It would be further complemented by systematic control of environmental and physiological variables such as clothing characteristics, protocol stimuli, and skin hydration status. Additionally, future studies could explore the incorporation of effective electrode impedance measurements to assess and mitigate variability introduced by differences in skin–electrode contact, hydration, and placement. This could provide a more objective means of ensuring signal quality across different body locations and experimental conditions.

## 5. Conclusions

The objective of this study was to evaluate the feasibility of alternative torso locations for EDA acquisition as part of a multimodal, belt-worn IBS monitoring device. Using a within-subject design, EDA signals were systematically collected from four distinct torso locations and compared to finger EDA while participants performed two different tasks: the Stroop and Cold Pressor tasks. The analysis focused on EDA arousal across tasks, with the meanSCL, NS.SCRs, and TVSymp as the primary outcome measures. Notably, the use of TVSymp in this context represents a novel approach, as, to the best of our knowledge, this is the first instance of its application in evaluating torso-specific locations. While further research is warranted using torso-specific devices and electrodes, the findings indicate that the torso, particularly the mid-chest or lower back, considering body shape limitations, may serve as a viable location for EDA recording due to its task sensitivity and close correlation with finger EDA throughout the protocol.

## Figures and Tables

**Table 1.** Comparison of EDA Response Across Locations.

		Location				
	Task	Fingers	Mid-Chest	Upper Abdomen	Lower Back	Mid-Back
***MeanSCL*** *(μS)*	*Baseline*	7.81 ± 3.48	6.00 ± 4.71	4.41 ± 3.95	6.90 ± 6.88	5.49 ± 4.57
		*[5.47, 10.1]*	*[3.16, 8.85]*	*[2.02, 6.80]*	*[2.93, 10.9]*	*[2.85, 8.13]*

	*Stroop*	9.79 ± 4.50	5.78 ± 5.19	5.22 ± 4.14	7.24 ± 6.84	3.83 ± 4.07
		*[6.76, 12.8]*	*[2.65, 8.92]*	*[2.72, 7.73]*	*[3.10, 11.4]*	*[1.48, 6.18]*
	*Cold Pressor*	10.4 ± 4.16	7.35 ± 5.50	6.05 ± 3.75	9.17 ± 8.07	5.93 ± 3.62
		*[7.42, 13.4]*	*[4.02, 10.7]*	*[3.53, 8.56]*	*[3.40, 14.9]*	*[3.74, 8.11]*

***NSSCR*** *(count/min)*	*Baseline*	57.1 ± 21.2	41.3 ± 20.8	45.5 ± 20.0	36.5 ± 24.1	44.2 ± 19.5
		*[42.8, 71.3]*	*[28.7, 53.8]*	*[33.4, 57.6]*	*[22.6, 50.4]*	*[32.9, 55.5]*

	*Stroop*	75.1 ± 16.8 *	45.7 ± 15.2	48.5 ± 16.7	41.9 ± 20.5	43.2 ± 19.3
		*[63.8, 86.4]*	*[36.6, 54.9]*	*[38.5, 58.6]*	*[29.5, 54.2]*	*[32.0, 54.3]*

	*Cold Pressor*	68.1 ± 16.8	50.7 ± 10.5	51.1 ± 20.7	42.0 ± 19.7	51.9 ± 12.3
		*[56.0, 80.1]*	*[44.4, 57.1]*	*[37.2, 65.0]*	*[27.9, 56.2]*	*[44.4, 59.3]*

***TVSymp*** *(n.u.)*	*Baseline*	0.255 ± 0.239	0.394 ± 0.366	0.319 ± 0.337	0.406 ± 0.430	0.385 ± 0.431
		*[0.094, 0.416]*	*[0.173, 0.615]*	*[0.115, 0.522]*	*[0.158, 0.654]*	*[0.136, 0.634]*

	*Stroop*	0.813 ± 0.447 *	0.617 ± 0.509	0.520 ± 0.395	0.577 ± 0.459	0.484 ± 0.459
		*[0.513, 1.11]*	*[0.309. 0.925]*	*[0.281, 0.759]*	*[0.300, 0.854]*	*[0.219, 0.748]*

	*Cold Pressor*	0.737 ± 0.435 *	0.878 ± 0.606 *	0.734 ± 0.629	0.718 ± 0.535	0.73 ± 0.552
		*[0.426, 1.048]*	*[0.512, 1.24]*	*[0.311, 1.16]*	*[0.336, 1.10]*	*[0.396, 1.06]*

Results for EDA indices organized by test and location. Displayed as statistical mean and standard deviation (mean ± std) [lower CI, upper CI].

(*) indicates statistical difference noted between Baseline and Stroop or Baseline and Cold Pressor tasks.

TVSymp, time-varying index of sympathetic skin conductance level; NS.SCRs, non-specific skin conductance responses; SCL, skin conductance level; n.u., normalized units.

Units as labeled.

**Table 2.** Correlation of Metrics Across Protocol Between Fingers and Test Locations.

Metric	Location			
	Mid-Chest	Upper Abdomen	Lower Back	Mid-Back
MeanSCL	0.58	0.96	0.78	−0.10
NSSCR	0.58	0.64	0.91	0.02
TVSymp	0.77	0.78	0.83	0.63

Reported as the correlation coefficient between the mean aggregate waveform for the labeled location and the fingers across all three phases of the study. No correlation demonstrated significance *p* < 0.05.

## Data Availability

The raw data and supplemental calculated metrics supporting the conclusions in this article will be made available by the authors upon request.

## Abbreviations

The following abbreviations are used in this manuscript:

EDA: Electrodermal Activity
IBS: Irritable Bowel Syndrome
meanSCL: Mean Skin Conductance Level
NS.SCR: Non-Specific Skin Conductance Response
TVSymp: Time-Varying Sympathetic Index
ECG: Electrocardiogram
EMG: Electromyogram
SNS: Sympathetic Nervous System
Ag/AgCl: Silver–Silver Chloride
